# Binary Classification Quantum Neural Network Model Based on Optimized Grover Algorithm

**DOI:** 10.3390/e24121783

**Published:** 2022-12-06

**Authors:** Wenlin Zhao, Yinuo Wang, Yingjie Qu, Hongyang Ma, Shumei Wang

**Affiliations:** 1 School of Information and Control Engineering, Qingdao University of Technology, Qingdao 266520, China; 2 School of Science, Qingdao University of Technology, Qingdao 266520, China

**Keywords:** binary classification, Grover algorithm, QNN

## Abstract

We focus on the problem that the Grover algorithm is not suitable for the completely unknown proportion of target solutions. Considering whether the existing quantum classifier used by the current quantum neural network (QNN) to complete the classification task can solve the problem of the classical classifier, this paper proposes a binary quantum neural network classifical model based on an optimized Grover algorithm based on partial diffusion. Trial and error is adopted to extend the partial diffusion quantum search algorithm with the known proportion of target solutions to the unknown state, and to apply the characteristics of the supervised learning of the quantum neural network to binary classify the classified data. Experiments show that the proposed method can effectively retrieve quantum states with similar features. The test accuracy of BQM retrieval under the depolarization noise at the 20th period can reach 97% when the depolarization rate is 0.1. It improves the retrieval accuracy by about 4% and 10% compared with MSE and BCE in the same environment.

## 1. Introduction

The Internet has many different kinds of data and information that are intersected and stored on social networks, prompting many different research fields to start to pay attention to social networks. Users on social networks obtain the resources that they need by visiting web pages [1,2,3,4]. The key to studying social networks is to analyze how these social networks are used. The data analyzed can be used to improve the social network itself, making it more convenient for users to browse the data required. They can also be used to analyze users’ preferences to deliver advertisements at designated points. They can also be used to analyze user behavior and to predict the transactions that users participate in [5,6]. One of the main ways to analyze these results is to perform extensive data analysis on the weblogs of these sites [7,8]. Every time a user requests a page or some of the resources on that page, such as video, sound, etc., a new record is added to the weblog of the site [9]. This information contains information about the user’s favourite pages (i.e., the most frequently visited pages), the sequence of visits to ordinary pages, and even hints at the user’s characteristics. This information analysis method can be called web page using data mining (WUM) based on weblog information [10,11,12]. To use WUM, a sequence of interactions between a single user and a web page needs to be extracted. The resulting file should contain at least the following fields: the user’s IP address, timestamp, requested resource, code for the result of the operation, the previous web address before entering the web page, and the browser used [13,14]. Using and analyzing this sequence, the pattern of the user’s access to the web page can be obtained.

The value of big data is essentially reflected as follows: it provides new thinking and means for a human to understand complex systems. In theory, a virtual digital image of the natural world can be constructed by digitizing the real world on a sufficiently small scale of time and space that carries the running rules of the real world. On the premise of sufficient computing power and efficient data analysis methods, an in-depth analysis of this virtual digital image will make it possible to understand and to discover the existing complex system’s operation behavior, state, and law. Big data provides a new way of thinking and a new means for exploring objective laws and for transforming nature and society for human beings. Due to the high and increasing demand for big data mining and analysis work, the era is forced to gradually use more efficient quantum scientific research technology [15] to meet the gap of technology improvement means, and then to improve the efficiency of information extraction work and to promote the progress of more scientific research. In view of the above requirements and improved thinking, this paper makes use of the special advantages of quantum algorithms compared with traditional iterative algorithms [16], as a new field of quantum neural network, and studies the neural network autonomous learning scheme [17] for network log information extraction, which highlights the high-value prospects of the quantum field in big data mining and analysis.

This search hassle can be carried out in O(N) with the use of Grover’s quantum search algorithm. Grover’s quantum search algorithm [18] makes use of the amplitude amplification approach in quantum computing to attain quadratic acceleration in unsorted search problems. Grover’s quantum search algorithm has been efficiently applied on classical computer systems with the usage of a quantum laptop language (QCL). For an unordered database search, the Grover algorithm achieves quadratic acceleration compared with the classical algorithm. Then, analysis, induction, and variant research are carried out [19,20]. Except for the lower bound, Grover’s methodology can be used for the case where the λ fraction of the target term is unknown. The fixed point strategy and the trial and error method are the two quantum search strategies that can be implemented.

The Binary QNN Model in this paper is a kind of model based on the Grover algorithm and the QNN supervised learning algorithm. First of all, this is based on the traditional Grover algorithm being analyzed and improved, using Younes’ algorithm to improve the search algorithm efficiency, inserting this into the iterative learning process of the quantum neural network [21,22], and using quantum processes to promote the efficiency of the algorithm and neural network to realize multiple network synchronization searching and learning [23,24] for each iteration algorithm to improve the efficiency of solutions [25]. The quantum neural network learning scheme in this paper can be applied to quickly find the user’s IP address in massive weblogs, and then accurately and efficiently identifying and classifying relevant and valuable information such as the IP addresses of network logs. The results can be sorted according to the number of search categories to identify and analyze user behavior accurately. It can not only quickly discover the person’s record of specific interests, but also can divide the session of a single user, which ensures the accuracy of person document identification and improves the effectiveness of user activity queries.

This article is structured as follows. Section 2 introduces the trial-and-error method (Younes’ algorithm) used in this paper, and the basic principles of the QNN supervised learning division task. Section 3 describes our binary QNN model. Section 4 describes the evolution process of the original dataset, and provides entropy analysis to show the advantages of this model. Section 5 summarizes and discusses the role of the proposed model in the development of user behavior pattern prediction.

## 2. Basic Conception

### 2.1. Supervised Learning Classification of QNN

Quantum Neural Network (QNN) is a new research field formed by the intersection of quantum physics, mathematics, computer science, information science, cognitive science, complexity science, and other disciplines. As the natural evolution of the traditional neural computing system, it makes full use of the great power of quantum computing to improve upon the information processing capacity of neural computing. By introducing the idea of the superposition of quantum states into the traditional feedforward neural network, QNN can train the quantum interval and quantify the uncertainty of the input data of training samples. Different data will map to different magnitudes. A multi-layer excitation function is used to increase the fuzziness of the network, and to improve the accuracy and certainty of network pattern recognition [26]. Therefore, the research on the quantum neural network provides beneficial support for the combination of quantum computing and neural computing. The potential of quantum neural networks is that they take advantage of both quantum computing based on coherent superposition and neural computing based on parallel processing. For example, running a deep neural network (DNN) model on a device with limited computing power can be challenging because of the large computational power and memory requirements on the device. However, to solve this problem, a quantum neural network (QNN) has greater potential, which can save computing costs while ensuring the accuracy of DNN training.

QNN comprises quantum state preparation subcircuits and optimization tasks performed by classical controllers [27]. The fact that variable-component subcircuits utilized in QNN produce probability distributions that cannot be efficiently simulated is part of the evidence supporting the claim [28,29]. QNN’s main application, similar to DNN’s, is to tackle categorization tasks [30]. Practical challenges, such as recognizing handwritten digits and the features of many living creatures, can be categorized as categorization scenes [31,32].

A dataset is given

(1) $$\begin{array}{c} T={\left\{\left(x_{i},y_{i}\right)\right\}}_{i=0}^{N-1}\in \left(R^{N\times M},{\left\{0,1\right\}}^{N}\right) \end{array}$$

**Figure 1:**
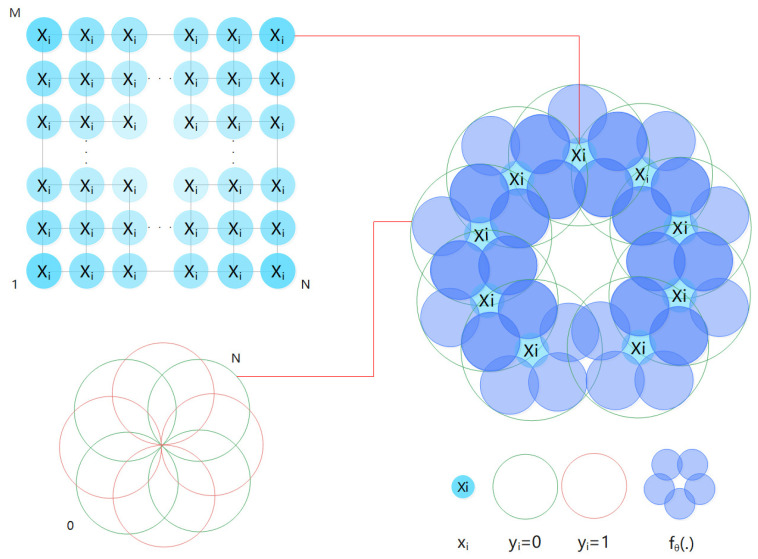
Basic principles of the quantum classifier. $x_{i}$ randomly generated by the $R^{N\times M}$ matrix, and $y_{i}$, which can only be 0 or 1, forms *N* data pairs and then generates the dataset *T*. According to the classic backpropagation rule $f_{\theta }\left(\cdot \right)$, $x_{i}$ is taken as the input to this rule. The qubit obtains the input–output relationship from the data pair $\left(x_{i},y_{i}\right)$ in the constructed dataset *T*, and learns the interaction in the training set of the relationship. In other words, the purpose of the quantum neural network is to use $x_{i}$ as an input to learn $f_{\theta }\left(\cdot \right)$ rules.

According to *N* examples and *M* elements in the examples, a QNN is led to research $f_{\theta }\left(\cdot \right)$ to predict the label of a facts set *T*

(2) $$\begin{array}{c} \min_{\theta }\sum_{i=0}^{N-1}I_{y_{i}\neq f_{\theta }\left(x_{i}\right)} \end{array}$$

where θ is the trainable parameter, and $I_{z}$ is an indicator function whose value is 1 when the condition *z* is met; otherwise, it is zero. The quantum classifier realizes the data tag prediction function according to specific rules through the filtered data, and its basic principle is shown in Figure 1. We use quantum classifiers in the research section to specify a QNN for completing the classification task defined in Formula (2). Considering the binary task, it is necessary not only to find a decision rule in Formula (2), but also to output the index *j* satisfying the pre-determined black box function. Given a trained classifier $f_{\theta }\left(\cdot \right)$, both classical algorithms and previous quantum classifiers require at least O(T) query complexity to find *j*.

The dataset *T* is constructed from a given qubit with adjustable interactions, where the qubit composition is represented by $x_{i}$ and $y_{i}$. We learn the interaction from a given training set of each input–output relationship based on the classical backpropagation rule $f_{\theta }\left(\cdot \right)$, and taking $x_{i}$ as the input to its rule, where the input–output relation is the data pair $\left(x_{i},y_{i}\right)$ that constitutes the dataset *T*. This learning process of qubits is viewed as the desired output algorithm behavior, that is, the quantum network “learns” an algorithm.

A notable theoretical result concerning quantum classifiers is the tradeoff between the computational cost and the training performance shown [33].

### 2.2. Younes’ Algorithm

The methodology presented by Younes, Rowe J., and Miller J. [34] in Younes’ algorithm is used to carry out the quantum search by exploiting the local diffusion operator to overcome the souffle problem in the Grover algorithm. It demonstrates that regardless of whether the number of matches is known, the entire range of 1≤M≤N can be consistently handled. It lays the theoretical foundation of the binary QNN model.

In the |0〉 and |1〉 states, part of the diffusion operator $Q_{i}$ system subspace of the entanglement in additional qubit workspace performs about the inverse operation of the mean, and the inverse operation of the phase shift is −1. *H* is the Hadamard Gates denoted by $H=\frac{1}{\sqrt{2}}\left[\begin{array}{cc} 1 & 1\\ 1 & -1 \end{array}\right]$. The diagonal representation of $Q_{i}$ applied to the n+1 qubit system is:

(3) $$\begin{array}{c} Q_{i}=\left(H^{⊗n}⊗I_{1}\right)(2|0\rangle \langle 0|-I_{n+1})\left(H^{⊗n}⊗I_{1}\right) \end{array}$$

asthe |0〉 length is $2^{n+1}$, $I_{1}$ is the unit of a 2×2 matrix.

Generally, quantum structures of the well-known size n+1 can be expressed as:

(4) $$\begin{array}{c} |\psi \rangle =\sum_{p=0}^{N-1}\alpha_{p}(|p\rangle ⊗\left|0\rangle \right)+\sum_{p=0}^{N-1}\beta_{p}(|p\rangle ⊗\left|1\rangle \right) \end{array}$$

Applying $Q_{i}$ to |ψ〉 bits, we obtain

(5) $$\begin{array}{c} \sum_{p=0}^{N-1}\left(\frac{2}{N}\sum_{p=0}^{N-1}\alpha_{p}-\alpha_{p}\right)(|p\rangle ⊗\left|0\rangle \right)-\sum_{p=0}^{N-1}\beta_{p}(|p\rangle ⊗\left|1\rangle \right) \end{array}$$

where $\frac{1}{N}\sum_{p=0}^{N-1}\alpha_{j}$ means the mean amplitude of subspace $\sum_{p=0}^{N-1}\alpha_{p}(|p\rangle ⊗\left|0\rangle \right)$. That is, the operator $Q_{i}$ only performs the inversion of the means in the subspace and only changes the sign of the amplitude.

The *H* gate is utilized to the first *n* qubits to produce $2^{n}$ values to characterize the list. Then, we iteratively observe the Oracle feature $U_{f}$ to map the goal in the list to 0 or 1, and we retail the outcomes such as $U_{f}|x,0\rangle \to |x,f\left(x\right)\rangle$; the partial diffusion operator $Q_{i}$ is applied, and this step is repeated *q* times. Finally, the first *n* qubits are measured.

The variety of iterations *q* has to be an integer to locate a healthy shut to the change in measurements.

Setting $q=\left\lfloor \frac{\pi }{2\theta }\right\rfloor$, as $|q-\bar{q}|\leq \frac{1}{2}$, $0<\theta \leq \frac{\pi }{2}$. As $\cos\left(\theta \right)=1-\frac{M}{N}$, we can find

(6) $$\begin{array}{c} \theta \geq \frac{\sqrt{2NM-M^{2}}}{N} \end{array}$$

(7) $$\begin{array}{c} q=\left\lfloor \frac{\pi }{2\theta }\right\rfloor \leq O\left(\sqrt{\frac{N}{M}}\right) \end{array}$$

It is proven that the algorithm can be handled in the range of 1≤M≤N using the $O(\sqrt{\frac{N}{M}})$ fixed operator.

## 3. The Binary QNN Model

**Figure 2:**
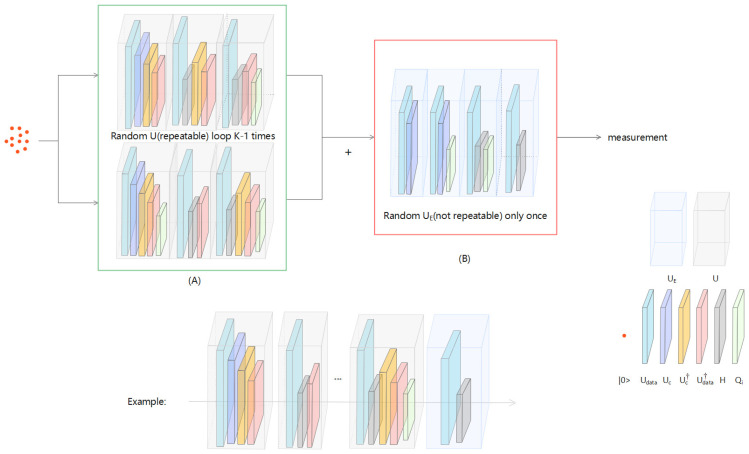
The paradigm of BQM. (**A**) The first K-1 loop uses *U*, defined in Equation (12), which consists of unitary operators (namely $U_{data}$, $U_{c}$, *H*, and $Q_{i}$). (**B**) The last cycle uses the unitary operation $U_{E}$ defined in Equation (13). The qubit interacts with $U_{c}$ and $Q_{i}$ to form the feature register and data register.

We simulate the creation of a binary analysis algorithm that uses quantum states to process information, as shown in Figure 2. The algorithm proposed in this paper is uniformly represented as a BQM field in the following content. As shown in Figure 2, BQM uses a specified variable component subcircuit $U_{c}$, and an *H* gate to replace the Oracle $U_{f}$. The variable component subcircuit $U_{c}$, based on the training data, can conditionally flip a flag qubit. The tagged qubit is then used as part of the *H* gate to guide a Grover search algorithm to identify the index of the specified example; i.e., the state of the tagged qubit, such as “0” or “1”, determines the probability of success in identifying the target index. BQM optimizes the trainable parameters of the variable component subcircuit $U_{c}$. When the corresponding training instance is positive, the success probability of sampling a target index is maximized. Otherwise, BQM minimizes the probability of the success of sampling target indicators. The design of our algorithm has some advantages in terms of query complexity by inheriting attributes from the Grover search algorithm and performing binary classification tasks on these attributes while allowing the setting of search constraints [35]. Under the above observation, a quantum classifier must have certain advantages [36,37,38].

### 3.1. Pretreatment Stage of a Dichotomous Task

In the pretreatment stage, a dichotomous task uses the dataset *T* defined in Equation (1) as the extended dataset $\hat{T}$.

To apply the Grover search algorithm to obtain index i=K−1, for K∈[N], the $K^{th}$ pair data training rules $T_{k}$ are as follows.

(8) $$\begin{array}{c} T_{K}{=[(x}_{k}^{\left(0\right)}{,y}_{k}^{\left(0\right)}{),(x}_{k}^{\left(1\right)}{,y}_{k}^{\left(1\right)}),\ldots {,(x}_{k}^{\left(K-1\right)}{,y}_{k}^{\left(K-1\right)})] \end{array}$$

The pair of data in $T_{k}$ is like the $K^{th}$ pair of $\hat{T}$; this means that $\left(x_{k}^{\left(K-1\right)},y_{k}^{\left(K-1\right)}\right)=\left(x_{k},y_{k}\right)$. The first K−1 pair of $T_{k}={\left\{\left(x_{k}^{\left(i\right)},y_{k}^{\left(i\right)}\right)\right\}}_{i=0}^{K-2}$ uniformly samples from a subset of $\hat{T}$, when every label ${\left\{y_{k}^{\left(i\right)}\right\}}_{i=0}^{K-2}$ is the opposite of $y_{k}$.

$y_{k}\in \left\{0,1\right\}$, ${\hat{T}}^{\left(0\right)}$ and ${\hat{T}}^{\left(1\right)}$ are constructed, which contain only those examples of $\hat{T}$ with the labels ‘0’ and ‘1’, respectively. When $y_{k}=0$, the pair samples before *K* from ${\hat{T}}^{\left(1\right)}$; it is same as for the situation where $y_{k}=1$, in which the pair samples before *K* from ${\hat{T}}^{\left(0\right)}$.

Various quantum classifiers encode $T_{k}$ into quantum states in different ways [39]. For the sake of notation, we indicate that $|\Phi^{k}\rangle$ that analogously connects with the $K^{th}$ example is

(9) $$\begin{array}{c} |\Phi^{k}\rangle =U_{data}|0\rangle =\sum_{i=0}^{K-1}\frac{1}{\sqrt{K}}|g\left(x_{i}\right)\rangle |i\rangle \end{array}$$

as g(·) is a coding operation.

### 3.2. The Training Process of the Learning Plan

Compared with the traditional Grover algorithm, combining the variational learning method and the Grover search algorithm produces quantum advantages [40,41,42,43,44]. The adopted variable component subcircuit $U_{c}$ is designed to find a hyperplane to keep the last pair in the $T_{k}$ away from the pair of samples before *K*.

For the variational quantum circuits $U_{c}\left(\theta \right)$ in BQM, a NISQ device scheme consists of a trainable single-qubit gate and two-qubit gates such as CNOT or CZ, which implement generation and discrimination tasks using variational hybrid quantum-classical algorithms. $U_{c}$ is denoted as $U_{c}=\prod_{c=1}^{C}U\left(\theta^{c}\right)$, where each layer $U(\theta^{c})$ contains O(poly(N)) parameterized single-qubit gates and at most, O(poly(N)) fixed two-qubit gates with the same layout.

In the optimal situation, given a initial state $|\Phi^{k}\rangle$ defined in Equation (9), $U_{c}$ is applied to obtain the following goals:

1. If the pair of samples before *K* as $T_{k}$ analogously connects with the label $y_{k}=0$, the expected state is
  (10) $$\begin{array}{c} \left(U_{c}⊗I\right)(U_{\mathrm{data}}|0\rangle {)}_{y_{k}=0}=\sum_{i=0}^{K-1}\frac{1}{\sqrt{K}}|0\rangle |i\rangle \end{array}$$
2. If the pair of samples before *K* as $T_{k}$ analogously connects with the label $y_{k}=1$, the expected state is
  (11) $$\begin{array}{c} \left(U_{c}⊗I\right)(U_{\mathrm{data}}|0\rangle {)}_{y_{k}=1}=\sum_{i=0}^{K-1}\frac{1}{\sqrt{K}}|1\rangle |i\rangle \end{array}$$

The label $y_{k}=0$ (or $y_{k}=1$) as the quantum state of the $R_{F}$ characteristics register the first qubits |0〉 (or |1〉). As shown in Figure 2A, state $\left(U_{c}⊗I_{I}\right)U_{data}|0\rangle$ was prepared. Our binary QNN model iteratively applied the H door to register by the characteristics of the first qubit and index on the index register control, using the $U_{data}$ register and the $U_{c}$ calculation characteristics, and to finish the first cycle, it applied the diffusion operator $Q_{i}$ to the index register. All quantum processes, such as *U*, are part of a period.

(12) $$\begin{array}{c} U=Q_{i}\circ U_{data}^{†}\circ {\left(U_{c}⊗I\right)}^{†}\circ H\circ \left(U_{c}⊗I\right)\circ U_{data}^{} \end{array}$$

Define $Q_{i}=I⊗\left(\frac{2}{K}\sum_{i}\left|i\rangle \langle i\right|-I_{I}\right)$. Except on a loop, the binary QNN model is repeatedly in the initial state |0〉 to *U*, and the application of the unitary operation is replaced with

(13) $$\begin{array}{c} U_{E}=Q_{i}\circ H\circ \left(U_{c}⊗I\right)\circ U_{data}^{} \end{array}$$

The brown shade in Figure 2B shows this. According to the traditional Grover search algorithm [45], before making quantum measurements, the binary QNN model polls *U* and $U_{E}$ for a total of $O(\sqrt{K})$ times.

### 3.3. The Evolution of the Quantum State

We analyze how quantum states evolve under $y_{k}=0$ and $y_{k}=1$:

- After interaction with unitary $U_{c}⊗I$, using the Equation (10) input state $\Phi_{k}\left(y_{k}=0\right)$, this state can be converted to $\frac{1}{\sqrt{K}}\sum_{i=0}|0\rangle |i\rangle$. For all computing in i∈[K−1], this means that the quantum operation $Q_{i}\circ U_{data}^{†}\circ {\left(U_{c}⊗I_{I}\right)}^{†}$ does not change state.
  (14) $$\begin{array}{c} \frac{1}{\sqrt{K}}\left(H^{⊗n}⊗I\right)(2|0\rangle \langle 0|-I_{n+1})\left(H^{⊗n}⊗I\right)\sum_{i=0}^{K-1}|0\rangle |i\rangle =\sum_{i=0}^{K-1}\frac{1}{\sqrt{K}}\left|0\rangle \right|i\rangle \end{array}$$
  When we measure the indicator register of the output state, the sampling i∈[K−1] for calculating the base *i* is distributed.
- After interaction with unitary $U_{c}⊗I$ using the Equation (11) input state $\Phi_{k}\left(y_{k}=1\right)$, this state can be converted to $\frac{1}{\sqrt{K}}\sum_{i=0}|1\rangle |i\rangle$.
  Mathematically, the result state is generated after interaction with *H*
  (15) $$\begin{array}{c} H\circ \left(U_{c}⊗I\right)(U_{data}|0\rangle {)}_{y_{k}=1}=\frac{1}{\sqrt{K}}|0\rangle \sum_{i=0}^{K-2}|i\rangle -\frac{1}{\sqrt{K}}\left|1\rangle \right|i^{*}\rangle \end{array}$$
  where $|i^{*}\rangle$ for calculating the base |K−1〉. The calculation operation $U_{data}^{†}\circ {\left(U_{c}⊗I\right)}^{†}$ and the diffusion operation $Q_{i}$ are used to increase $|i^{*}\rangle$ probability.
  After the first cycle, the generated state is generated
  (16) $$\begin{array}{c} U(U_{\mathrm{data}}|0\rangle {)}_{y_{k}=1}=\frac{\left(K-4\right)}{K\sqrt{K}}|0\rangle \sum_{i=0}^{K-2}|i\rangle +\frac{3K-4}{K\sqrt{K}}\left|0\rangle \right|i^{*}\rangle \end{array}$$
  where Equation (12) defines *U*. According to Grover’s algorithm, the chance of sampling $i^{*}$ will increase to $\frac{{\left(3K-4\right)}^{2}}{K^{3}}$.

### 3.4. The Loss Function

With the observation above leading to Theorem 1, the proof is given above:

**Theorem** **1.** For BQM, under the optimal setting, if the label of the last item of $T_{k}$ is $y_{k}=1$, the probability of the sampling resulting in $i^{*}=K-1$ is asymptotically 1.

**Proof** **of** **Theorem** **1.** We discussed the case where the last entry in $T_{k}$ has labels $y_{k}=1$ and $y_{k}=0$.

In the instance of $y_{k}=0$, assuming that the label of the final item in $T_{k}$ is $y_{k}=0$, it is possible to determine from Equation (14) that after the first cycle, the generation state of BQM is

(17) $$\begin{array}{c} U_{c}\left|\Phi_{k}\left(y_{k}=0\right)\rangle =U\right|0\rangle =\sum_{i=0}^{K-1}\frac{1}{\sqrt{K}}\left|0\rangle \right|i\rangle \end{array}$$

The chance of picking any index when *U* (apply to |0〉) is the same, according to the formula above. After *U* is applied to |0〉 via induction, with the migration with time *n*, the state changes as

(18) $$\begin{array}{c} \prod_{i=0}^{N}U^{i}|0\rangle =\sum_{i=0}^{K-1}\frac{1}{\sqrt{K}}\left|0\rangle \right|i\rangle \end{array}$$

where the given *N* is any positive integer, and the probability of sampling $|i^{*}\rangle$ is $\frac{1}{K}$. In the last loop, the quantum operation $U_{E}$ defined in Equation (13) is applied to state $\prod_{i=0}^{N}U^{i}|0\rangle$ and the resulting state is

(19) $$\begin{array}{cc} U_{E}\prod_{i=0}^{N}U^{i}|0\rangle & =Q_{i}\circ H\circ \left(U_{c}⊗I\right)\circ U_{data}^{}\sum_{i=0}^{K-1}\frac{1}{\sqrt{K}}|0\rangle |i\rangle \\ \; & =\sum_{i=0}^{K-1}\frac{1}{\sqrt{K}}|0\rangle \Lambda \end{array}$$

where the first equality uses Equation (18); the second equation uses Equation (15) and exploits the application of the diffusion operator $Q_{i}=\left(H^{⊗n}⊗I_{1}\right)(2|0\rangle \langle 0|-I_{n+1})\left(H^{⊗n}⊗I_{1}\right)$, then $\Lambda =\frac{1}{\sqrt{K-1}}\sum_{i=0}^{K-2}|i\rangle +|i^{*}\rangle$.

In the instance of $y_{k}=1$, assuming that the label of the last item in $T_{k}$ is $y_{k}=1$, it is possible to determine from Equation (16) that after the first cycle, the generation state of BQM is

(20) $$\begin{array}{c} U_{c}|\Phi_{k}\left(y_{k}=1\right)\rangle =|0\rangle ⊗(\frac{\left(K-4\right)}{K\sqrt{K}}\sum_{i=0}^{K-2}|i\rangle +\frac{3K-4}{K\sqrt{K}}|i^{*}\rangle ) \end{array}$$

The chance of picking any index when *U* (apply to |0〉) is the same, according to the formula above. After *U* is applied to |0〉 by induction, with the migration with time *n*, the state changes as follows:

(21) $$\begin{array}{c} \prod_{i=0}^{n}U^{i}|0\rangle =|0\rangle ⊗\frac{1}{\sqrt{K}}\left[\hbar \sum_{i=0}^{K-2}|i\rangle +\;\lambda |i^{*}\rangle \right] \end{array}$$

where given that *n* is any positive integer, $\hbar =\cos\left(2n\alpha \right)-\frac{1}{\sqrt{K-1}}\sin\left(2n\alpha \right)$, $\sin\alpha =\frac{1}{\sqrt{K}}$, $\;\lambda =\sqrt{K-1}\sin\left(2n\alpha \right)+\cos\left(2n\alpha \right)$. In the last loop, the quantum operation $U_{E}$ defined in Equation (13) is applied to state $\prod_{i=0}^{N}U^{i}|0\rangle$, and the resulting state is

(22) UE∏i=0NUi|0〉=Qi∘H∘(Uc⊗I)∘Udata|0〉⊗1Kℏ∑i=0K−2|i〉+λ|i*〉)=Qiℏ|0〉∑i=0K−2|i〉+λ|1〉|i*〉K=(K−2)ℏ|0〉∑i=0K−2|i〉+2K−1λ|1〉|i*〉K3

where the first equality uses Equation (21) and the second equation uses Equation (16) to design the feature register. It uses *H* to flip the phase of |i〉 whose first qubit of the feature register is |1〉, and the last equation comes from the application of the diffusion operator $Q_{i}$.

According to Equation (22), in the ideal situation, the probability of sampling $i^{*}$ is near to 1 when $n∼O(\sqrt{K})$, and then $\left(K-1\right)\sin\left(2n\alpha \right)+\sqrt{K-1}\cos\left(2n\alpha \right)$ is close to 1.

The result of Equation (19) shows that when $y_{k}=0$, the probability of sampling $i^{*}$ never increases. Thus, we can follow that the sampling probability of the result $i^{*}$ asymptotically approaches one if and only if the label of the last term of $T_{k}$ is $y_{k}=1$. □

According to Theorem 1 of the BQM’s special property, the output distribution is different for different labels of the input $T_{k}$ while performing the binary classification task. According to the analysis of Theorem 1 mentioned above, the calculation basis i=K−1 will be present in the output state of the BQM; that is, $U_{E}U^{O(\sqrt{K})}|0\rangle$, which corresponds to $y_{k}=1$, and its probability is close to 1. The matching output state for $y_{k}=0$ will, however, include the same computational foundation i∈[K−1].

According to the mechanism of the Grover search algorithm, the loss function of BQM is deduced as

(23) $$\begin{array}{c} \min_{\theta }L\left(\theta \right)=s\left(\frac{1}{2}-y_{k}\right)Tr\left((|1\rangle \langle 1|)⊗H⊗(|i^{*}\rangle \langle i^{*}|)\Delta_{\theta }\right) \end{array}$$

where s(·) is the sign function, $\Delta_{\theta }=U_{E}U{\left(\theta \right)}^{O(\sqrt{K})}\left|0\rangle \langle 0\right|{\left(U_{E}U{\left(\theta \right)}^{O(\sqrt{K})}\right)}^{†}$, and U(θ) is defined in Equation (12).

The success probability of sampling $i^{*}$ and obtaining the first feature qubit as ’1’(’0’) is maximized (minimized) when $y_{k}=1$ ($y_{k}=0$), when faced with the challenge of minimizing the loss function L(θ).

### 3.5. Gradient-Based Parameter Optimization

The optimization method in this paper uses a multiple-layer parameterized quantum circuit (MPQC), according to the principle that the arrangement of quantum gates in each layer is the same [46], and the operation formed by the layer *c* is expressed as $U(\theta^{c})$, produced by quantum states produced by MPQC

(24) $$\begin{array}{c} |\omega \rangle =\prod_{c=1}^{C}U\left(\theta^{c}\right)|0\rangle^{⊗N} \end{array}$$

where *C* is the total number of layers. BQM uses MPQC to construct $U_{c}$

(25) $$\begin{array}{c} U_{c}\left(\theta \right)=\prod_{c=1}^{C}U(\theta^{c}) \end{array}$$

**Figure 3:**
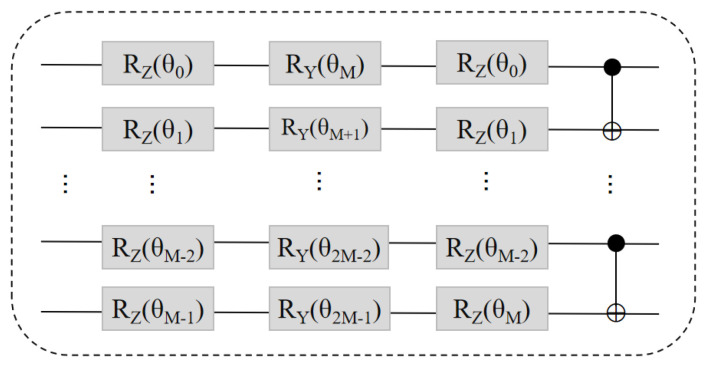
The realization of the $l^{th}$ layer $U(\theta^{c})$. It is assumed that the $l^{th}$ layer $U(\theta^{c})$ interacts with *M* qubits. Three trainable parameterized gates $R_{Z}$, $R_{Y}$, and $R_{Z}$ are first applied to each qubit, followed by the M−1 CNOT gates.

The circuit layout of $U(\theta^{c})$ at layer *l* is shown in Figure 3. When the number of layers is *C*, the total number of trainable parameters of BQM is 2MC.

The update rules of BQM at the $j^{th}$ iteration are as follows

(26) $$\begin{array}{c} \theta^{\left(j+1\right)}=\theta^{\left(j\right)}-\zeta \frac{L(\theta^{\left(j\right)},T_{j})}{\partial \theta } \end{array}$$

where ζ is the learning rate. Given the explicit form of L(θ) in the defined Equation (23), the gradient of $L(\theta^{\left(j\right)},T_{j})$ can be rewritten as

(27) $$\begin{array}{c} \frac{\partial L(\theta^{\left(j\right)},T_{j})}{\partial \theta }=s\left(\frac{1}{2}-y_{j}\right)\frac{\partial Tr(\prod \Delta_{\theta^{\left(j\right)}})}{\partial \theta } \end{array}$$

where $y_{j}$ is the label of the last item in $T_{j}$, s(·) is the symbol function, and ∏ is the measurement operator.

BQM employs a gradient-based method, according to the parameter displacement rule, to obtain the gradient $\frac{\partial Tr(\prod \Delta_{\theta^{\left(j\right)}})}{\partial \theta }$, to optimize θ. The parameter shift rule [47] iteratively calculates each gradient entry under its guiding principle.

For e∈[2NC], only the $e^{th}$ parameter is rotated by $\pm \frac{\pi }{2}$, i.e.,

(28) $$\begin{array}{c} \theta_{\pm }^{\left(j\right)}=\left[\theta_{0}^{\left(j\right)},\ldots ,\theta_{e-1}^{\left(j\right)},\theta_{e}^{\left(j\right)}\pm \frac{\pi }{2},\theta_{e+1}^{\left(j\right)},\ldots ,\theta_{2NC-1}^{\left(j\right)}\right] \end{array}$$

Combining Equations (26)–(28), the update rule of BQM at the $e^{th}$ iteration of the e item is

(29) $$\begin{array}{c} \theta_{e}^{\left(j+1\right)}=\theta_{e}^{j}-\zeta s\left(\frac{1}{2}-y_{j}\right)\frac{\partial Tr(\prod \Delta_{\theta_{\pm }^{\left(j\right)}})}{2} \end{array}$$

where $\Delta_{\theta_{\pm }^{\left(j\right)}}=U_{E}U{\left({\theta_{+}}^{\left(j\right)}\right)}^{O(\sqrt{K})}\left|0\rangle \langle 0\right|{\left(U_{E}U{\left({\theta_{-}}^{\left(j\right)}\right)}^{O(\sqrt{K})}\right)}^{†}$.

### 3.6. Circuit Implementation of Label Prediction

**Figure 4:**
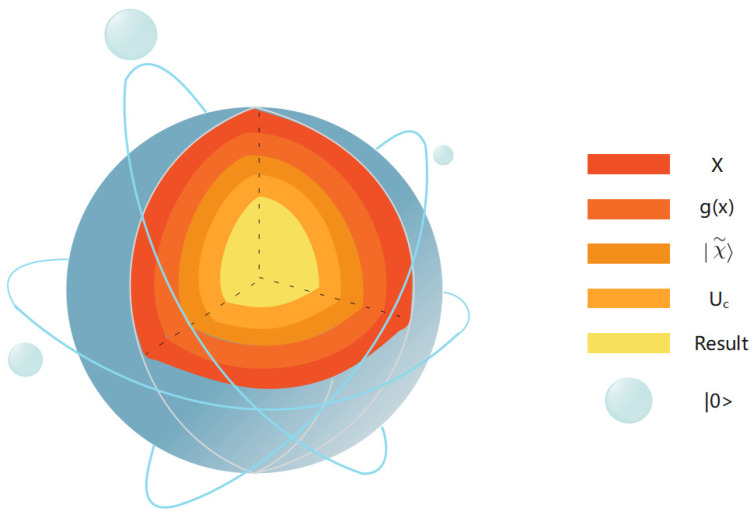
Circuit implementation of BQM prediction. Use g(·) as in the training process. The encoding method prepares the state |g(x)〉 and applies the trained variable component subcircuit $U_{c}$ to $|\tilde{\chi }\rangle$.

After the training of BQM is completed, the trained $U_{c}$ can use the corresponding circuit (as shown in Figure 4) to predict the label of an instance with O(1) query complexity.

Denoting the new input as (x,y), we encode *a* into quantum states using the same encoding method used during training; i.e., $|\tilde{\chi }\rangle =|g\left(x\right)\rangle$, then we apply the trained $U_{c}$ to $|\tilde{\chi }\rangle$.

When the size of the dataset loaded by the binary QNN model is *K*, a well-trained binary QNN model can obtain the index with $O(\sqrt{\frac{K}{MT^{2}}})$ query complexity.

### 3.7. Synthetic Construction of Datasets

**Figure 5:**
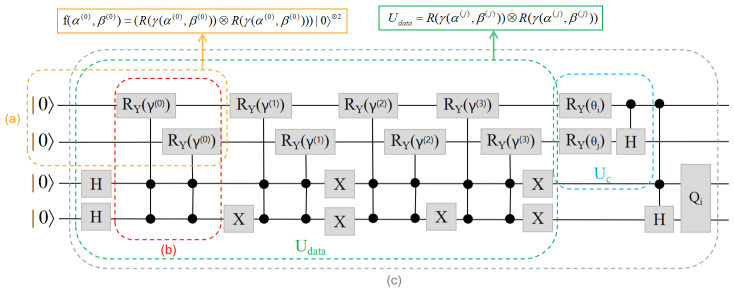
Implementation of BQM in numerical simulation. (**a**) The circuit implementation of the encoded unitary $U_{data}$ corresponding to the feature map $f(x_{i})$ is illustrated. (**b**) The realization of quantum operation $f(x_{i})$. (**c**) The implementation of BQM, given input $T_{k}=\left\{x_{i},x_{j},x_{m},x_{n}\right\}$.

Given the training example $x_{i}=\left(\alpha^{\left(j\right)},\beta^{\left(j\right)}\right)\in R^{2}$, the embedded function $f(\alpha^{\left(j\right)},\beta^{\left(j\right)})$ used to encode $x_{i}$ into a quantum state is represented as

(30) $$\begin{array}{c} f\left(\alpha^{\left(j\right)},\beta^{\left(j\right)}\right)=\left(R\left(\gamma \left(\alpha^{\left(j\right)},\beta^{\left(j\right)}\right)\right)⊗R\left(\gamma \left(\alpha^{\left(j\right)},\beta^{\left(j\right)}\right)\right)\right)|0\rangle^{⊗2} \end{array}$$

where $\gamma \left(\alpha^{\left(j\right)},\beta^{\left(j\right)}\right)={\left(\alpha^{\left(j\right)},\beta^{\left(j\right)}\right)}^{2}$ is a specified mapping function. The above formula means that g (xi) can be converted into a series of quantum operations, the implementation of which is shown in Figure 5a. To encode multiple training examples into quantum states simultaneously, we should treat $f(x_{i})$ as a controlled version, the implementation of which is shown in Figure 5b.

### 3.8. The Details of BQM

The implementation of GBLS is shown in Figure 5c. In it, the data encoding the unitary $U_{data}$ consists of a controlled set of $f(x_{i})$ quantum operations. The implementation of encoding unity $U_{data}$ depends on the size of the batch B. For the quantum kernel classifier with BCE loss and MSE loss (B=M), it can be seen from Formula (30) that the unitary encoding is

(31) $$\begin{array}{c} U_{data}=R\left(\gamma \left(\alpha^{\left(j\right)},\beta^{\left(j\right)}\right)\right)⊗R\left(\gamma \left(\alpha^{\left(j\right)},\beta^{\left(j\right)}\right)\right) \end{array}$$

For a quantum kernel classifier with MSE loss (B=M/4), the implementation of encoding unitary $U_{data}$ is the same as that of BQM, as shown in Figure 5.

## 4. Results

We will analyze the security of the proposed BQM, intuitively express the evolution and generation process of the dataset by using the dot plot, and evaluate the algorithm’s performance through entropy analysis and testing [48,49,50].

### 4.1. Dataset Evolution

**Figure 6:**
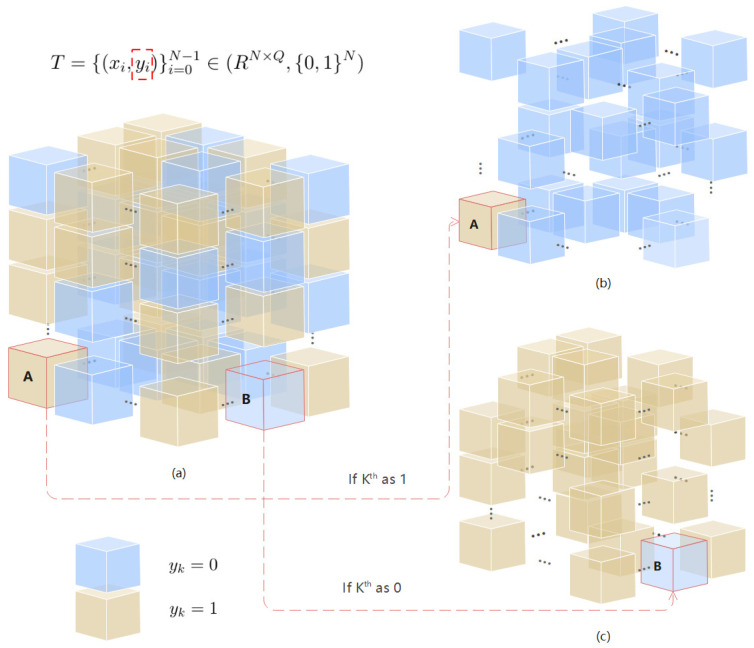
Dataset evolution. The yellow cube in the figure represents the data point in the data pair whose y=1, the blue cube represents the data point in the data pair whose y=0, and the point circle in red represents the $K^{th}$ data point. (**a**) The traditional dataset when defined as *T*, which is defined in Equation (1). (**b**) When $K^{th}=1$ (that is, the red grid labeled A), according to the generation formula, $K^{th}$ is combined with the first K−1 labels with y=0, and the resulting dataset. (**c**) When $K^{th}=0$ (that is, the red grid labeled B), according to the generation formula, $K^{th}$ is combined with the first K−1 labels with y=1, and the resulting dataset.

This algorithm’s dataset generation process is shown in Figure 6. It uses the top K−1 pair and the $K^{th}$ pair in the original dataset for label classification and uniform sampling. It divides all data pairs into sub-datasets labeled as 1 (or 0) according to the *y* value of 0 (or 1).

The yellow cube in the figure represents the data point in the data pair whose value of *y* is 1, the blue cube represents the data point in the data pair whose value of *y* is 0, and the point whose circle in red represents the $K^{th}$ data point. The specific rules are as follows:

1. Represents the original dataset when $y_{k}=1$ as the `A’ cube or when $y_{k}=0$ as the `B’ cube in the $K^{th}$ pair of data $\left(x_{k},y_{k}\right)$;
2. Represents the uniform sampling from the sub-dataset labeled 1 in Figure 6a to generate a new dataset in Figure 6b;
3. Represents the uniform sampling from the sub-dataset labeled 0 in Figure 6a to generate a new dataset in Figure 6c.

### 4.2. Stability and Convergence Analysis

Define a utility-bound R as a utility measure to evaluate the distance between the optimization result and the stationary point in the optimized environment.

(32) $$\begin{array}{c} R=E[||\nabla_{\theta }L\left(\theta^{\left(j\right)}\right){\left||\right]}^{2}\leq \varepsilon \left(j\right) \end{array}$$

For the BQM quantum classifier with a depolarization noise setting, the utility bound of output $\theta^{\left(j\right)}\in R^{l}$ after *j* iterations is

(33) $$\begin{array}{c} \varepsilon \left(j\right)=O(poly\left(\frac{l}{j{\left(1-P\right)}^{d}},\frac{l}{BK{\left(1-P\right)}^{d}},\frac{l}{{\left(1-P\right)}^{d}}\right)) \end{array}$$

where *P* is the depolarization rate, *l* is the total number of trainable parameters, *K* is the number of measurements to estimate the quantum expected value, *d* is the circuit depth of the variable component sub-circuit, and *B* is the number of batches.

We use the decay rate of log(ε(j)) to define the asymptotic convergence rate of this optimization algorithm [51,52]. According to Equation (33), the attenuation rate of log(ε(j)) is slower than that of −j, which proves that this algorithm has a sublinear convergence rate.

When B=M, we input each sample $T_{j}$ in turn to variable component subcircuits to obtain $\nabla L(\theta ,T_{j})$. Once the set ${\left\{\nabla L\left(\theta ,T_{j}\right)\right\}}_{j=1}^{M}$ is collected, the gradient ∇L(θ,T) can be estimated by $\frac{1}{M}\sum_{j=1}^{M}\nabla L(\theta ,T_{j})$. Assuming that the number of measurements required to estimate the derivative of the JTH parameter $\theta_{j}$ is *K*, the total number of measurements obtained is MK for $\frac{1}{M}\sum_{j=1}^{M}\nabla L(\theta ,T_{j})$. Therefore, the estimate of $\nabla L(\theta ,T_{j})$ with *l* parameters requires MKl measurements.

For the above definition of utility bound *R*, the results show that a large number of lot *B* can guarantee a better realization of utility bound *R* by increasing the total number of measurements.

### 4.3. Performance Analysis under Depolarization Noise

We employ depolarization channels to mimic the system noise, since the number of measurements and the quantum system’s noise are both constrained. We next examine how well BQM performs in the presence of depolarization noise [53].

If a quantum state is ω, we define the depolarizing channel $\varpi_{P}$ acting on this quantum state as

(34) $$\varpi_{P}\left(\omega \right)=\left(1-P\right)\omega +P\frac{I_{l}}{l}$$

where *P* is the depolarization rate, and *l* is the total number of trainable parameters.

**Figure 7:**
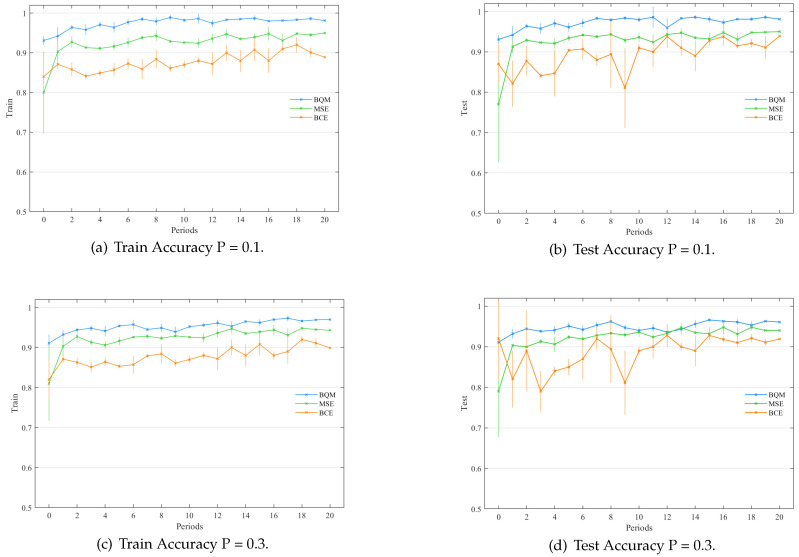
The performance of different quantum classifiers at the different depolarization rates (P=0.1,0.3). Depolarizing noise models extracted from quantum hardware are applied to the trainable unitary $U_{c}\left(\theta \right)$ of these three classifiers. The labels `BQM’, `BCE’, and `MSE’ refer to the proposed Grover-based quantum classifier, the quantum kernel classifier with BCE loss, and the quantum kernel classifier with mean square error loss. (**a**,**b**) shows the variation of the train and test accuracies of BQM and the quantum kernel classifier with BCE loss with a *P* value of 0.1. (**c**,**d**) show the variation of the train and test accuracies of BQM and the quantum kernel classifier with BCE loss when the *P* value is 0.3. Vertical bars reflect the variance of the train and test accuracy at each iteration.

We compare the performance of BQM and two other quantum kernel classifiers when quantum system noise and measurement delays are considered. Among them, BQM stands for a binary classification quantum neural network model based on the optimized Grover algorithm proposed in this paper. The two classifiers compared with BQM are defined as “BCE” and “MSE”, respectively. “BCE” stands for the quantum kernel classifier with binary cross-entropy loss, and “MSE” means the quantum kernel classifier with the mean square error loss (B = N). We simulated the statistics for each of the three classifiers by repeating the values 10 times. Figure 7 illustrates the simulation findings. After 20 periods, BQM, BCE, and MSE quantum classifiers achieve the same performances. It can be observed that the quantum classifiers with MSE loss have lower convergence speeds and larger variances than the BQM and BCE classifiers. This phenomenon reflects that using BQM for classification tasks with different batches is meaningful. In Table 1, we compare the average training and testing accuracies of the BQM, BCE, and MSE quantum classifiers in the last stage. Considering the measurement error and quantum gate noise, BQM still achieves a stable performance because of its minimal variance.

The binary QNN model based on Grover, quantum kernel classifier BCE loss, and quantum kernel classifier mean square error loss is reflected by the labels `BQM’, `BCE’, and `MSE’. The train and test accuracies of the BQM quantum classifier are shown in the left and right figures. The vertical bar represents the train and test accuracy variation at each iteration, where all hyperparameter settings are the same as those used in the above numerical simulation.

According to the numerical simulation results of the three quantum classifiers in Figure 7, BQM can obtain a good utility boundary R through some tests. When they achieve basically comparable performances, BQM reduces the number of measurements required by K = 4 times compared to quantum classifiers with BCE losses and MSE losses (B = N). This result shows that when N is larger, there is a large separation of computational efficiency between BQM and the previous B = N quantum classifier.

The above data demonstrate that, when BQM is compared to the other two quantum classifiers, the number of measurements required by BQM is decreased by four times, demonstrating BQM’s efficacy.

### 4.4. Complex Comparsion Analysis

**Figure 8:**
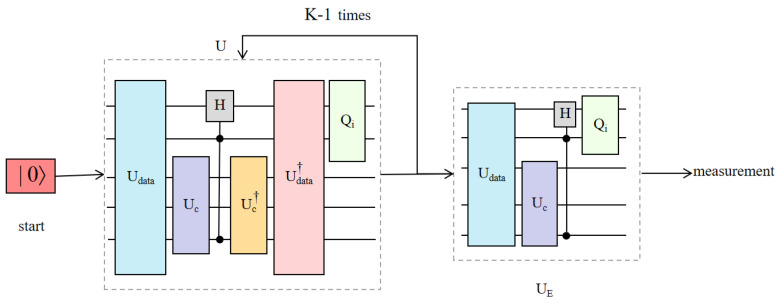
The iterated process of BQM algorithm. After receiving the encoded data in the variable component subcircuit of the quantum classifier, the measurement operator defines a query as one measurement.

After receiving the encoded data in the variable component subcircuit of the quantum classifier, the measurement operator defines a query as one measurement. The iterative process of this algorithm is shown in Figure 8. According to the quantum classifier’s training mechanism, calculating the total number of measurements for variable component subcircuits is comparable to the query complexity of obtaining the gradient in a time frame.

The next step is to derive the number of measurements required for a quantum kernel classifier with BCE loss in one period. For the dataset *T*, the BCE loss is generated

(35) $$L_{\mathrm{BCE}}=-\frac{1}{N}\sum_{i=0}^{N-1}y_{i}\log\left(P\left(y_{i}\right)\right)+\left(1-y_{i}\right)\log\left(1-P\left(y_{i}\right)\right)$$

where $y_{i}$ is the label of the $i^{th}$ example, and $P(y_{i})$ is the prediction probability of the label $y_{i}$; the output of its quantum circuit is

(36) $$P\left(y_{i}\right)=Tr((|1\rangle \langle 1|)⊗H⊗\nabla$$

where $\nabla_{\theta }=|i^{*}\rangle \langle i^{*}|)U_{c}\left(\theta \right)\left|0\rangle \langle 0\right|U_{c}{\left(\theta \right)}^{†}$, $U_{c}\left(\theta \right)$ is defined in Equation (25), and $(|1\rangle \langle 1|)⊗H⊗(|i^{*}\rangle \langle i^{*}|)=\prod$ is the measurement operator. According to the parameter displacement rule, the derivative of BCE loss is satisfied

(37) $$\frac{\partial L_{\mathrm{BCE}}}{\partial \theta_{e}}=\frac{1}{N}\sum_{i=0}^{N-1}\rho \frac{\mathrm{Tr}\left(\prod \nabla_{\theta_{+}}\right)-\mathrm{Tr}\left(\prod \nabla_{\theta_{-}}\right)}{2}$$

where $\theta_{\pm }$ is defined in Equation (28), $\rho =\frac{1-y_{i}}{1-P\left(y_{i}\right)}-\frac{y_{i}}{P\left(y_{i}\right)}$. To obtain the gradient of BCE loss according to the above equation, we need to give each training example to the quantum kernel classifier to estimate $P(y_{i})$, and then calculate the coefficient ρ.

BQM uses the superposition property of the loss function *L* defined in Equation (17) to obtain the gradient $\frac{\partial L}{\partial \theta_{e}}$. According to Equation (23), the gradient of BQM satisfies

(38) $$\frac{\partial L\left(\theta ,T_{k}\right)}{\partial \theta_{e}}=\frac{s}{2}\left(1-y_{k}(\mathrm{Tr}\left(\prod \nabla_{\theta_{+}^{\left(k\right)}}\right)-\mathrm{Tr}\left(\prod \nabla_{\theta_{-}^{\left(k\right)}}\right))\right)$$

where $y_{k}$ refers to the label of the last pair in the training example $T_{k}$. The gradient of $T_{k}$ may be calculated using 2K measurements, where the first *K* measurement aims to approach $\mathrm{Tr}\left(\prod \nabla_{\theta_{-}^{\left(k\right)}}\right)$ and the last *K* measurement aims to approximate $\mathrm{Tr}\left(\prod \nabla_{\theta_{+}^{\left(k\right)}}\right)$, according to the equation above.

Complex comparison analysis determines the effect of the dataset size on the binary QNN model in this paper. The standard Grover search algorithm’s search complexity is $O(\sqrt{Kl})$ for information data entries of size *K* and the total number of trainable parameters *l*. The optimal algorithm classification complexity value is $O(\sqrt{\frac{Kl}{MT^{2}}})$, as can be shown in the following Table 2. The reduced query complexity of BQM implies a potential quantum advantage in completing the classification task.

## 5. Conclusions

As an essential source of information for value-added social media websites, user behavior patterns are the key to fast and accurate identification through session division to realize extensive data analysis. In this paper, based on the trial-and-error method of the Grover search algorithm, combined with the binary classification task of QNN supervised learning, the advantages of the Grover quantum algorithm are brought into play in the quantum classifier of the quantum neural network. The data are preprocessed by analyzing the network search data to realize the construction of the BQM algorithm. They lay the foundation for the development of user behavior pattern prediction. The experimental data show that the application effect of this algorithm has a more apparent accurate recognition rate than the other two classifiers, and it still has a prominent effect in the depolarized noise environment. It can play a supervisory role in the security detection of future users’ network search behaviors.

## Figures and Tables

**Table 1:** The average training and testing accuracies of BQM, BCE, and MSE quantum classifiers in the last stage. The value `a ± b’ means that the average precision is a and its variance is b. The labels `BQM’, `BCE’, and `MSE’ refer to the proposed Grover-based quantum classifier, the quantum kernel classifier with BCE loss, and the quantum kernel classifier with mean square error loss.

Algorithm’s Name	P=0.1 (Train)	P=0.1 (Test)	P=0.3 (Train)	P=0.3 (Test)
BCE	0.883±0.034	0.871±0.071	0.924±0.042	0.799±0.061
MSE	0.941±0.017	0.938±0.008	0.929±0.034	0.917±0.011
BQM	0.977±0.011	0.971±0.007	0.951±0.027	0.949±0.010

**Table 2:** Query complexity in several algorithms. The notations T, K, M, and l refer to the batch size range, the wide variety of measurements used to estimate quantum expectation value, the complete variety of education examples, and the total number of trainable parameters.

Algorithm’s Name	Query Complexity
Grover	$O(\sqrt{Kl})$
Younes’ algorithm	$O(\sqrt{\frac{Kl}{M}})$
BCE	O(KMl)
MSE	O(KMl)
BQM	$O(\sqrt{\frac{Kl}{MT^{2}}})$

## Data Availability

The data are contained within the article.
